# Under Control? Mouse Models of Heat Exposure: A Systematic Review of Experimental Design and Methodology

**DOI:** 10.1111/apha.70281

**Published:** 2026-07-23

**Authors:** Amina H. Rhaman, Leaf R. Kardol, Shane K. Maloney, Ebony Quintrell, Elizabeth Sorial, Shannon Morgan, Danielle J. Russell, Erin Kelty, Caitlin S. Wyrwoll

**Affiliations:** ^1^ School of Population & Global Health, University of Western Australia Perth Western Australia Australia; ^2^ School of Human Sciences, University of Western Australia Perth Western Australia Australia; ^3^ The Kids Research Institute Australia Nedlands Western Australia Australia; ^4^ Medical School, University of Western Australia Perth Western Australia Australia

**Keywords:** experimental design, heat exposure, mouse models, systematic review, thermal stress, thermoregulation

## Abstract

Mouse models are widely used to study heat‐related physiological responses as they provide insights relevant to human health. However, species differences (including nocturnal behavior) and standard housing below the thermoneutral zone (TNZ; 26°C–34°C) can confound comparisons. This systematic review evaluates methodologies, including housing temperatures, used in mouse heat‐exposure studies published over the past 4 years. Medline, Web of Science, and Embase were searched for peer‐reviewed mouse heat‐exposure studies published from 2020 to 10 January 2025. Data on housing temperature, heat‐exposure protocols, and physiological outcomes were extracted. Screening and extraction were conducted independently and in duplicate in Covidence. One hundred seventy studies were included. Of those, 142 (83%) housed control mice below TNZ; seven (4%) studies maintained TNZ conditions and the remainder did not report control temperature. Heat exposures ranged from 30°C–45°C, most commonly 39°C–41°C. Body temperature was measured in 110 (65%) studies, often targeting ~42°C. When timing was reported, exposures occurred mainly during the light phase (52, 30%). Most studies used males only (116, 68%); 31 (18%) used females only; 9 (5%) included both sexes; and 14 (8%) did not report sex. Common outcomes were digestive (55, 32%), inflammatory (47, 28%), and central nervous system (40, 24%). Recent mouse heat‐exposure studies often compare heat‐exposed animals with cold‐stressed controls and apply daytime exposures that conflict with the nocturnal behavior of mice and human diurnal pattern. Incomplete reporting of housing conditions limits interpretation. Future research should account for differences between mice and humans to improve translational relevance.

**Trial Registration:** PROSPERO: CRD42024611316

## 1 Introduction

Heat stress is the leading cause of weather‐related death, and climate change is causing a global rise in ambient temperature, with extreme heat events becoming increasingly severe, frequent, and longer lasting [1]. The resulting increase in heat exposure threatens human health, with heat stress known to contribute to all‐cause mortality as well as mortality and morbidity due to cardiovascular and respiratory disease, while exacerbating noncommunicable and infectious diseases [1]. However, the effect of exposure to extreme heat on physiological systems remains incompletely understood [2] and what is known is heavily informed by experimental models, including mice [3, 4, 5].

Mice can provide valuable insight into the physiological effects of heat exposure in humans, informing human heat‐health concerns spanning heatwaves, occupational and exertional exposures, and heat exposure during pregnancy and early life. However, there are substantial differences in body size, composition, and shape between mice and humans, which markedly influence thermal homeostasis, with mice exhibiting higher thermal conductance, more variable core temperature, a preference for warmer temperatures, and elevated metabolic rate compared with humans [4, 6, 7]. Further, humans seek thermoneutrality through clothing and climate‐controlled environments. Thus, experiments must be carefully designed to account for specific differences between the species [4]. The housing temperature of control mice is a key experimental consideration, because the maintenance of animals at thermoneutrality is essential to prevent cold‐induced thermogenic responses that would otherwise alter baseline metabolic and physiological measures, and thereby confound comparisons with heat‐exposed mice [7]. Housing temperature should be guided by the thermoneutral zone (TNZ), defined as the range of ambient temperature within which an animal can maintain core body temperature without additional metabolic or evaporative effort [8]. For a dressed human, the TNZ is approximately 18°C to 22°C (26°C–33°C for an undressed human), whereas for mice it is considerably higher, at 26°C to 34°C [9, 10, 11]. The TNZ and thermoregulatory thresholds also differ between the sexes in both humans and rodents [12, 13].

The National Research Council (NRC, 2011) recommends that laboratory mice be housed at 20°C–26°C [14]. In practice, many animal facilities set the temperature at the lower end of that recommended range to facilitate human comfort [15]. Thus, experimental mice are typically housed at temperatures below the TNZ for mice, which imposes chronic cold stress and leads to increased heat production to balance heat loss [9, 16]. The exposure to conditions below the TNZ can have profound effects on physiology and behavior, including increased food intake (~40% higher at 22°C vs. 30°C), increased body mass, and hypertrophy of the liver, kidneys, and heart [17]. Other physiological parameters, such as heart rate, sleep architecture, and arterial blood pressure, are also altered under cold‐housing conditions compared to the TNZ [18]. These changes raise concerns about the validity of the control group that is used to assess the effect of heat exposure because mice housed below the TNZ are not in a neutral physiological state but are undergoing stress‐induced thermogenesis [11]. It is not possible, then, to conclude whether any change that is observed during exposure to heat is because of the heat, per se, or because of the removal of cold stress.

Given the use of mouse models to study heat‐related physiological effects and the influence of methodological variables, it is essential to evaluate current practice. We hypothesized that contemporary studies of mouse heat‐exposure often house control animals below the TNZ, incompletely report housing conditions, and overlook nocturnal activity patterns, thereby biasing estimates of the effect of heat and limiting translational relevance. To address this, we reviewed key aspects of experimental design, including the timing and duration of heat exposure, the physiological systems examined, the associated health considerations, and the sex of the animals that were studied. We aimed to perform a systematic review of contemporary experiments on heat stress in mice to quantify the temperature of control housing relative to the TNZ; characterize the regimen for heat and body temperature measurement; assess the reporting of humidity, housing density, strain/sex, and light–dark phase; and appraise the risk of bias. Rather than synthesizing health outcomes, our goal was to evaluate current practice and provide actionable recommendations to improve the rigor and translational validity of studies on heat‐exposed mice.

## 2 Methods

**FIGURE 1:**
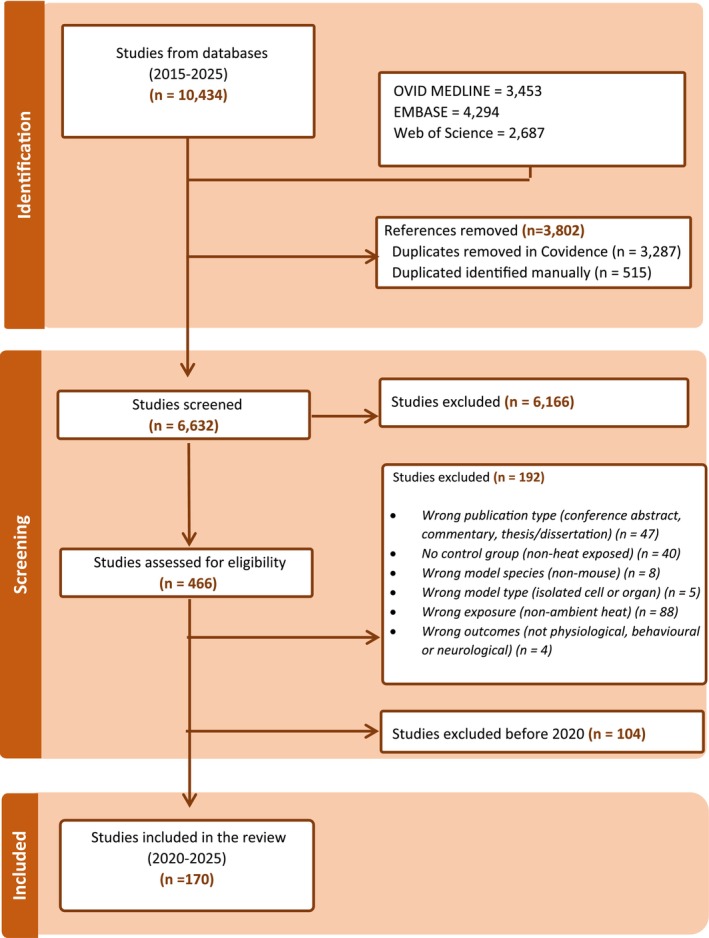
PRISMA diagram.

This review was prospectively registered on PROSPERO on December 5, 2024, and reported in accordance with the Preferred Reporting Items for Systematic Reviews and Meta‐Analyses (PRISMA) guidelines [19]. The original protocol included studies published between January 2015 and January 2025. However, the search yielded a large volume of eligible records (*n* = 267), an unfeasible workload for detailed methodological assessment. To focus on contemporary practices and maintain feasibility, we amended the eligibility criteria during data extraction to include only studies published from January 2020 to January 2025. This post hoc amendment is reported transparently and reflected in Figure 1.

### 2.1 Inclusion and Exclusion Criteria

Studies were included if they were original research articles involving mice ( *Mus musculus* ) exposed to high ambient temperatures, modeling heat stress or heat stroke, compared with a control group not exposed to high ambient temperatures, and assessing physiological, behavioral, or neurological outcomes (Data S1). Studies were excluded if the model was an isolated cell or organ rather than a mouse; focused on low ambient temperature; included solely exercise‐related elevations in body temperature, induction of fever, or a hot water bath; or lacked a relevant control group (Data S1).

### 2.2 Search Strategy, Screening and Data Extractions

Searches were conducted on January 10, 2025, using the MEDLINE, Web of Science, and Embase databases. Database‐specific search terms and headings for the components “mice” and “heat” were used (Data S1). Search results were uploaded to Covidence systematic review software, where de‐duplication, title and abstract screening, full‐text screening, and extraction were performed. Two reviewers independently screened and extracted each article, and any conflicts were resolved through discussion.

Data that were extracted included author and year of publication, country of publication, journal of publication, research institution, funding source, aim of the study, strain of mice studied, exposure details including the characteristics of housing temperature for control and heat exposed groups, exposure duration, timing of exposure, relative humidity levels, sample size per group, bodily system studied, effects of heat exposure, confounding factors, follow‐up duration, and study limitations. When the temperature and relative humidity of the housing were reported, we calculated the water vapor pressure (WVP) of the air [20]. Non‐English articles were translated using Google Translate (https://translate.google.com/). Additionally, the quality of each study was assessed using a checklist adapted from the CAMARADES checklist [21], which assessed whether each study had been published in a peer‐reviewed journal, had random allocation to treatment or control, had a blinded assessment of outcome, had done a sample size calculation, had complied with animal welfare regulations, and had a statement of potential conflicts of interest.

### 2.3 Data Synthesis

Given that the purpose of this review was to evaluate current practices rather than synthesize all available evidence on heat exposure, a meta‐analysis was not conducted. Instead, data were synthesized descriptively. We calculated counts and percentages to summarize the number of studies that housed control mice below, within, or above the TNZ. Where temperature ranges were reported, the midpoint of the range was used for analysis and graphing. We also quantified the proportion of contemporary studies that adhered to basic reporting standards for ambient humidity and other housing conditions. Six criteria were used to assess the risk of bias and reporting quality: peer‐review status, random allocation, blinding, sample‐size reporting, compliance with animal‐welfare regulations, and conflict‐of‐interest declarations. Data were exported to Microsoft Excel (Data S2).

## 3 Results

The search identified 6632 studies, of which 443 were screened in full text, and 170 were included in the final review (PRISMA flow diagram in Figure 1). There was a gradual increase in the number of studies each year, from 26 in 2020 to 59 in 2024. In 2025, only three studies were identified during the January search.

The studies included in this review were conducted in 12 countries. The majority originated from China (68%), followed by the United States (15%), and Japan (5%). Iran contributed six studies (4%), while Brazil, South Korea, Taiwan, Australia, and Canada each contributed two studies (1.2% each). Single studies came from Germany, India, and Israel (< 1% each).

A range of outcomes was investigated, with some studies investigating multiple outcomes. The most frequently examined outcomes were related to the digestive system (*n* = 55), inflammation (*n* = 47), and the central nervous system (CNS) (*n* = 40), followed by outcomes specifically related to the liver (*n* = 29) and behavioral outcomes (*n* = 23). Other commonly assessed areas included immunological (*n* = 17), respiratory (*n* = 16), hematological (*n* = 16), muscular (*n* = 15), pregnancy (*n* = 14), and cardiovascular (*n* = 14) outcomes. Less frequently studied outcomes included metabolism (*n* = 8), male reproduction (*n* = 7), female reproduction (*n* = 6), mortality (*n* = 5), and lactation (*n* = 4). Furthermore, studies examined articular cartilage (*n* = 1), growth (*n* = 1), the skeletal system (*n* = 1), and sleep (*n* = 1).

Sample size and sex varied widely across studies. Sample size typically ranged from 6 to 12 mice per experimental group, though the exact numbers varied considerably. Regarding sex, most studies focused on male mice (116 studies, 68%), with seven of those focusing on the male reproductive system. Thirty‐one studies (10%) focused solely on female mice, and most of those addressed the female reproductive context and pregnancy (20 studies, 67%). Further, nine studies (5%) included both sexes. Sex was not specified in 14 studies (18%).

Nine different mouse strains were used across the 170 studies. The most common strain was C57BL/6 (65%), followed by ICR (12%), BALB/c (8%), Kunming (7%), Swiss mice (4%), and CD‐1, NMRI, and ZCK mice (each < 1%). Six studies (3.5%) did not specify the mouse strain that was used.

### 3.1 Temperature and Humidity of the Control Housing

**FIGURE 2:**
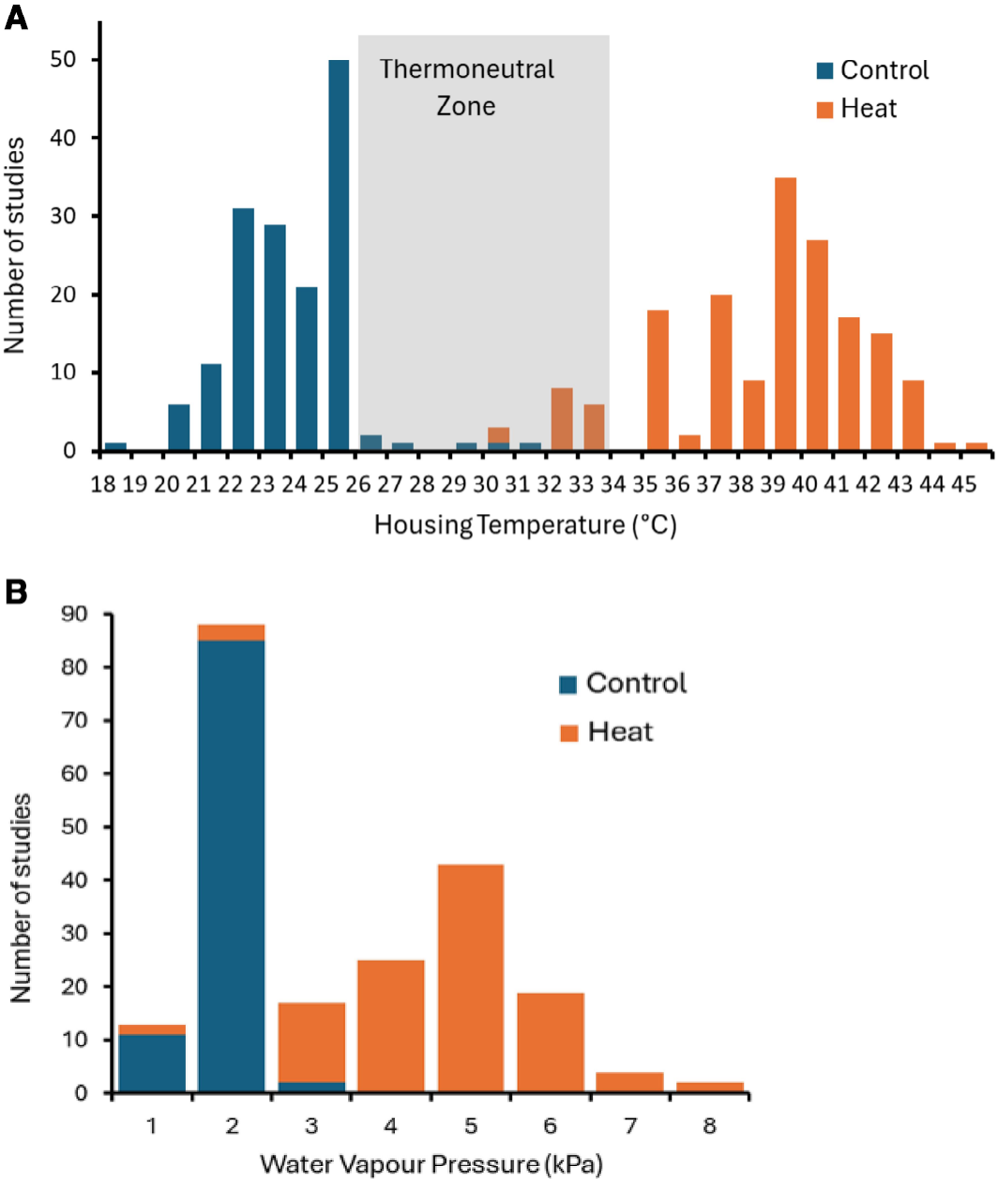
Distribution of ambient temperatures (A) and water vapor pressures (B) in the housing for control and heat‐exposed mice.

The ambient temperature of the housing for control mice varied from 18°C to 30°C. Most control groups (142 studies, 83%) were housed below the mouse TNZ, with temperatures ranging from 18°C to 25°C (Figure 2A). Only seven studies (4%) housed their control groups within the TNZ; the rest (5%) did not clearly specify the temperature of the control‐housing. Additionally, two studies used ambiguous descriptions (e.g., “ambient environment”). When humidity was reported, it was most often given as relative humidity. The humidity that the control group was exposed to was not reported in 71 studies (42%); among the 99 that did, values ranged from 22% to 77% relative humidity, most commonly 50% (27 studies), followed by 60% (14 studies), 55% (12 studies), and 35% (10 studies). In the 98 studies that reported both temperature and relative humidity for the control group, the WVP ranged from 0.66 to 2.4 kPa, with most studies reporting values between 1 and 2 kPa (Figure 2B).

When control housing temperatures were stratified by the sex of the animals that were studied, the distribution was similar across groups (Figure S1). Male‐only studies (*n* = 107) used control temperatures that ranged from 18°C to 27°C, and female‐only studies ranged slightly higher from 20°C to 31°C. Studies that included both sexes (*n* = 9) ranged from 20°C to 24°C. Only two studies in the entire dataset housed the control animals within the TNZ, both of which were female‐only studies (one at 29°C, one at 31°C).

### 3.2 Temperature and Humidity During Heat Exposure

The ambient temperature of the housing for heat‐exposed mice ranged from 30°C to 45°C. The most frequently reported temperature was 39°C (39 studies, 23%), followed by 40°C (26 studies, 15%), 37°C (20 studies, 12%), and 35°C (18 studies, 11%) (Figure 2). A small proportion (16 studies, 9%) exposed the mice to a temperature that was within the TNZ (≈26°C–34°C) but referred to the condition as heat exposure. The humidity for the heat‐exposed group was not reported in 56 studies (33%); among the 114 that did report humidity, values ranged from 10% to 99% relative humidity, most commonly 60% relative humidity (44 studies), followed by 50% relative humidity (11 studies), 55% relative humidity (eight studies), and 70% relative humidity (six studies). In the 113 studies that reported both temperature and relative humidity for the heat‐exposed group, the WVP ranged from 0.82 to 7.7 kPa, with most studies reporting values between 4 and 5 kPa (Figure 2B).

### 3.3 Body Temperature Measures of Control and Heat‐Exposed Groups

The methods that were used to measure body temperature varied across the 110 studies that reported body temperature. The most frequently reported approach was the use of a rectal probe (63 studies), while three additional studies combined measurements by rectal probe with measurements of body‐surface temperature. Telemetry was reported in 29 studies, with the measurement device implanted into the abdominal cavity, and two studies combined telemetry with measurements of body‐surface temperature. Body surface temperature alone was measured in two studies. Only a single study used oral temperature as a measure of body temperature.

**FIGURE 3:**
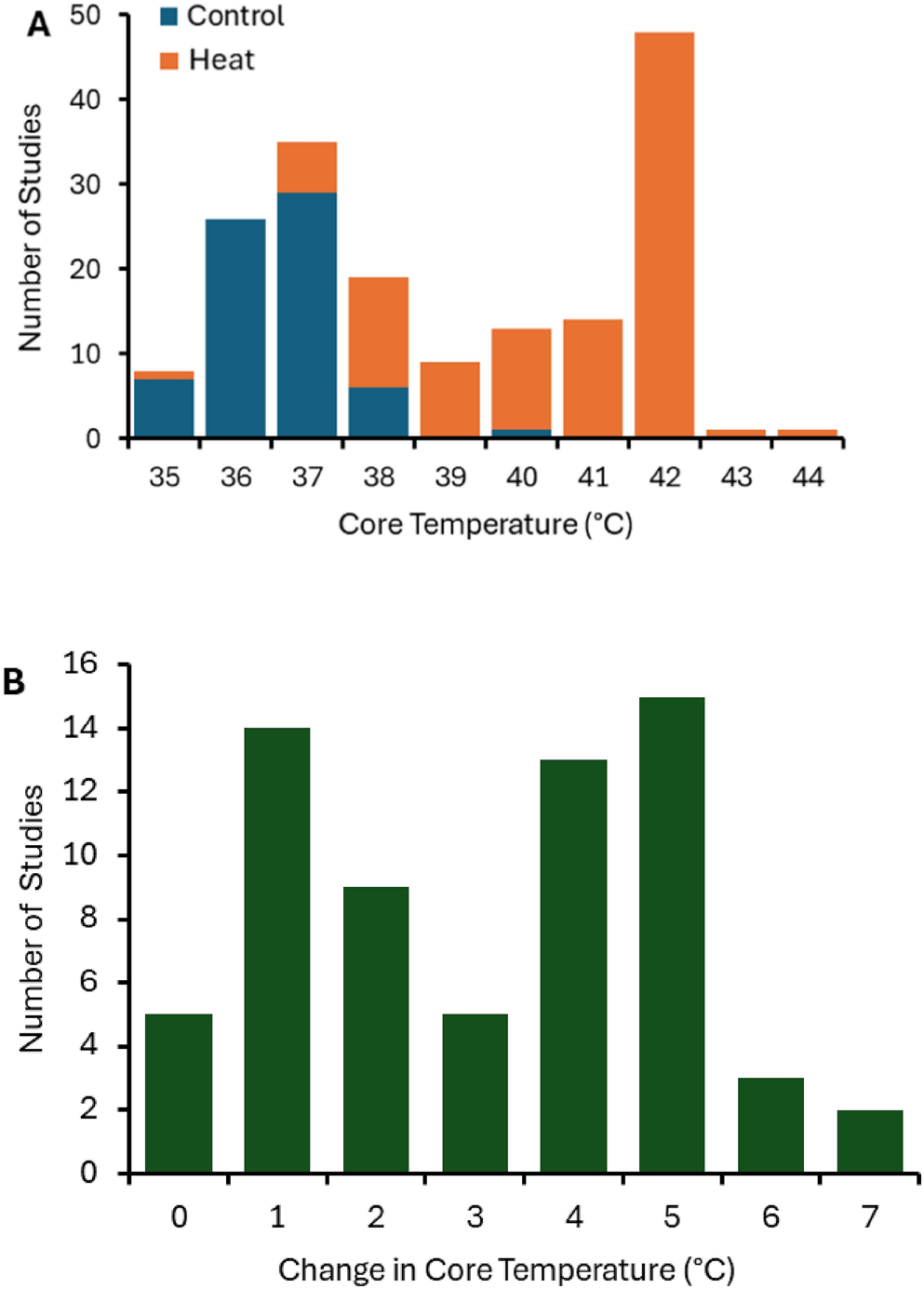
Distribution of core body temperature for control and heat‐exposed groups (A) and distribution of the change in core body temperature observed after heat exposure (B). Body temperature measurements include rectal probe measurements and intraperitoneal telemetry methods.

Body temperature was reported less frequently in the control groups than in the heat‐exposed groups. Of the 170 studies, only 73 (40%) reported body temperature in the control animals (Figure 3A). The reported values averaged 36.9°C, ranging from 35.1°C to 40.0°C, reflecting the effect of exercise prior to heat exposure. The body temperature of mice exposed to heat was reported in 110 studies (65%), with values ranging from 35.9°C to 43.0°C (Figure 3A). The most frequently reported temperature was 42°C (50 studies), with values ranging from 42.0°C to 42.9°C. This was followed by 41°C (11 studies), 40°C (10 studies), 39°C (nine studies), 37°C (six studies), 38°C (five studies), 35°C (1 study), and 43°C (one study).

In 66 studies (39% of the total), the temperatures of both the control and heat‐exposed animals were reported, allowing calculation of the temperature rise due to heat exposure (Figure 3B). The mean increase was 3.5°C, ranging from 0.2°C to 7.3°C. Notably, 47 studies reported an increase of ≥ 2°C, reflecting the emphasis on extreme heat stress in many models.

### 3.4 Regimen for Heat‐Exposure and Timing During the Light–Dark Phase

The duration of heat exposure and frequency were heterogeneous, ranging from brief single exposures (10 to 60 min) to repeated routines (1 to 12 h/day for 1 to 60 days), including some regimens of continuous exposure for 24 to 45 days. Some heat regimens modeled heat exhaustion, which entailed exposure until core temperature reached ~41°C–42.7°C or until collapse (43 studies). Further, 94 of the 170 studies that were included in this review (55%) did not report whether the heat intervention occurred during the light or dark phase. Among the 76 studies that did report the timing of the exposure, it occurred in the light phase in 52 studies, in the dark phase in three studies, and in both phases in 21 studies.

### 3.5 Other Housing Conditions

The number of mice that were housed per cage was reported in 46 studies (27%), most of which reported 1 mouse per cage (29 studies). Group housing was less common, with two studies housing two or three mice per cage, and one study housing either four mice or four to five mice per cage. One study described the mice as “co‐housed” without specifying the number. Apart from the number of animals per cage, reports on housing conditions beyond the mention of standard housing requirements, such as bedding type or whether enrichment was provided, were quite limited.

### 3.6 Factors Other Than Heat and Populations That Were Assessed

Experimental contexts were heterogeneous: while 32 studies examined the effect of heat alone, most combined heat with another factor. Those other factors included drugs/supplementation/nutrition (80 studies), exercise (12 studies), genetic modification (12 studies), and other environmental exposures (nine studies). Other factors included aging, immune function, or disease states. There was a small number of studies on microbiome‐related interventions and nanoparticles, and one on obesity.

### 3.7 Risk of Bias and Reporting Quality

Random allocation was reported in 108 studies (61%), while blinded assessment of outcomes was reported in only 29 studies (17%). A sample size calculation was reported in nine studies (5%). A statement of compliance with animal welfare regulation was included in 159 studies (94%), and a declaration of any conflicts of interest was provided in 160 studies (94%).

## 4 Discussion

This systematic review of studies that investigated the responses of mice to heat exposure highlights a couple of critical methodological concerns. Foremost, most of the control animals in studies of heat exposure were housed below the TNZ. Only 4% of the studies in the past 5 years housed their control animals within the TNZ. This sub‐thermoneutral housing can induce physiological cold stress, which will confound any interpretation of heat‐induced effects [11]. Furthermore, most studies exposed mice to heat during the light phase, when mice are typically asleep, creating a circadian mismatch for translation to humans, for whom ambient temperature usually peaks during the daytime awake period. When housing conditions were reported, including cage density and humidity, that reporting was inconsistent, emphasizing a need for more standardization in experimental reporting. Many studies focused on the effects of heat in otherwise healthy mice and predominantly investigated the male response. Further, studies frequently modeled prolonged or severe heat exposure that do not clearly reflect typical human exposure. A smaller subset explored potential therapeutic interventions or examined responses in models that represented at risk health conditions. Overall, current experimental models of heat exposure in mice have substantial limitations, and caution is warranted when the findings are extrapolated to human health considerations [11, 15, 22, 23].

### 4.1 The Problem With “Standard” Conditions: Temperatures Below Thermoneutrality

The apparently common practice to house mice at sub‐thermoneutral temperature raises methodological and translational concerns. First, when they are given a choice, mice consistently prefer environments that are within their TNZ [23, 24]. This preference reflects the cold stress that is experienced when mice are exposed to conditions that are below their TNZ [22], which alters physiology and behavior [17, 18]. Consequently, “heat‐exposed” mice are often compared to “controls” that are cold‐stressed. As such, these comparisons (between heat‐stressed and cold‐stressed mice) do not truly isolate the effects of heat.

The reason that the control conditions matter is that a mouse that is housed below its TNZ is not in a neutral physiological state—it is in a state of sustained cold‐induced thermogenesis. Brown adipose tissue is chronically activated; the drive on the sympathetic nervous system is elevated; food intake is roughly 40% higher than at thermoneutrality; body mass and the size of the liver, kidneys, and heart are increased; heart rate is elevated under sustained sympathetic stimulation as is blood pressure; and immune and inflammatory tone are shifted [11, 16, 17, 18, 24, 25]. The fundamental interpretive problem this creates is that when a cold‐housed mouse is exposed to heat, any change that is observed in the heat‐exposed group could reflect either the effect of heat itself, the removal of the cold‐driven responses, or both. There is no way to distinguish between these processes from the data alone. The only way to isolate the effect of heat is to begin from a thermally neutral baseline that is neither cold nor hot, so that whatever change is observed can be attributed to the heat exposure itself.

Heart rate is a clear example of a physiological parameter that can be misinterpreted if control mice are not housed under thermoneutral conditions. In mice housed at standard laboratory temperature (around 22°C), heart rate is high (approximately 600 beats per minute) [26]. However, this is not the mouse's resting heart rate; it is elevated because this temperature forces the mice to generate extra heat. When mice are instead housed at thermoneutral conditions (around 30°C), this cold‐driven thermogenesis is no longer needed, and heart rate falls to approximately 350–400 beats per minute [26]. Consistent with this, a study that housed control mice at sub‐thermoneutral temperatures (24°C) found that heat exposure (35°C) lowered heart rate [27]. These results suggest that heat exposure slows the heart, but the decrease in heart rate was, in fact, due to the withdrawal of cold‐driven thermogenesis. Without a thermoneutral baseline, the reported effect of heat can be wrong.

It has been argued that cold‐stressed mice (kept at ~20°C–22°C) provide translational relevance to humans because they align the metabolic rate (~1.6 × BMR at 20°C–22°C) with that of humans (~1.6 × BMR) [16]. However, in humans, this level of energy expenditure (~1.6 × BMR) occurs under thermoneutral conditions and is driven by activity [11]. In contrast, in cold‐stressed mice, the same metabolic rate (~1.6 × BMR) is driven by cold‐stress‐induced thermogenesis [11]. Much of that extra heat generation in cold‐exposed mice occurs in brown adipose tissue [28], while heat generation from human activity occurs in skeletal muscle. The metabolic and endocrine changes that are induced by the two types of activity are not the same [29]. Moreover, the argument that mice should be cold‐stressed to match the human basal metabolic rate has been challenged by original research that showed that mice housed at thermoneutral temperature exhibit a mean daily energy expenditure of 1.8 × BMR, a level that closely mirrors the pattern of human energy expenditure [30]. These findings call into question the justification to house control mice at anything other than their TNZ and instead support the routine use of thermoneutral housing.

### 4.2 What Temperature Should Mice Be Housed at?

There are various recommendations for the optimal temperature for housing mice within their TNZ. For example, Gordon et al. [31] recommend 30°C as optimal for mice, whereas Keijer et al. [22] argue that 30°C is too high, and instead propose a range of 25.5°C–27.6°C. In contrast, recent work suggests a “thermoneutral point” of 29°C during the resting (light) phase and ~33°C during the active (dark) phase [32, 33]. The combination of different recommendations with the wide range of thermoneutral values makes it difficult to determine a “right” temperature. As such, the optimal point within the TNZ can vary with the experimental context [24]. For example, lactating female mice have been shown to prefer lower ambient temperatures [34]. In contrast, tumor‐bearing mice tend to gravitate toward higher ambient temperatures (around 28°C–30°C) [35]. Thus, researchers should consult the relevant literature to determine the most appropriate thermal condition for their specific population, experimental group, or condition.

While in principle, a tailoring of the housing temperature to each specific experimental context would be ideal, such a nuanced approach is vague and would likely be impractical [36]. In standard animal facilities, multiple studies are often conducted in the same room, and so are all done at a single fixed temperature. The alternative would be dedicated climate chambers that would allow individualized temperature settings, and while that technology is available, it is not routine for most research facilities. Furthermore, the balance between experimental validity and staff comfort has to be considered. The maintenance of animal rooms at the upper end of the mouse TNZ (around 30°C or above) can be uncomfortable for personnel and challenging to sustain. Given these constraints, guidelines suggest that an ambient temperature of 26°C offers an optimal compromise between the thermoregulatory needs of mice and the need for staff to work there [23].

Notably, our analysis of control housing temperatures for the two sexes revealed that female‐only studies used a very similar range of ambient temperatures to that of male‐only studies, despite established sex differences in the TNZ and thermoregulatory responses in mammals [13, 37]. There were only two sex‐specific studies that housed controls at thermoneutral temperatures, which were studies on female mice. The consideration of sex is important given the growing recognition of sex as a biological variable in preclinical research and in thermoregulatory responses.

### 4.3 Timing of Heat Exposures: Circadian Impacts

More than half of the studies (55%) in this review did not report whether heat exposure occurred during the light phase or the dark phase. This is a significant omission given that there are circadian influences on thermoregulation, metabolism, and behavior [38]. Among studies that did report the timing of heat exposure, most applied heat during the light phase. Because mice are nocturnal, the exposure does not align with their natural active period. If the outcome of a study concerned the natural history of mice, then daytime exposure would be appropriate because that is what mice will generally experience in the natural environment. But for translational relevance, heat exposure should be administered during the dark phase, when mice are usually awake, metabolically active, and behaviorally engaged. This timing better mirrors human physiology and behavior during daytime heat exposure. Conversely, exposure to heat during the light phase in mice corresponds to overnight heat exposure in sleeping humans, a scenario of considerable public health importance given that hot nights prevent the nocturnal recovery of core temperature and are strongly associated with heatwave mortality. Studies that adopt either phase of exposure should specify and justify the translational scenario that is being modeled [39].

We found that fewer than one‐third of studies reported the number of mice per cage, and when they did, most experiments used single‐housed mice. That practice has important welfare, physiological, and thermoregulatory implications, since laboratory mice are inherently social animals [40, 41, 42]. Social isolation is thought to function as a stressor that can adversely impact physiological and metabolic parameters [42]. Further, because mice use behavioral adaptations to cold, including huddling, the TNZ of single‐housed mice is shifted up relative to that for group‐housed mice [31, 43]. Because most papers did not report the details of housing density, it is difficult to know the extent to which housing density may have confounded the reported physiological outcomes.

More than a third of the papers did not report the humidity that the mice were exposed to in either the heat‐exposed or control arms. For all homeothermic mammals, including mice, humidity is an essential variable because when the ambient temperature approaches or exceeds the body temperature, evaporative heat loss becomes the main (then only) route for heat loss, and the rate of evaporation decreases when the humidity increases [44, 45]. Because humidity can determine thermoregulatory and physiological outcomes, accurate reporting of humidity, ideally as the water vapor pressure because the relative humidity varies inversely with the air temperature at a given water vapor pressure, is essential for reproducibility and interpretation [44, 45]. Because the saturation vapor pressure increases exponentially with air temperature, relative humidity can be misleading regarding an animal's capacity to lose heat by evaporation. For example, it is possible for an animal to lose heat evaporatively into saturated air, that is, when the relative humidity is 100%, when the WVP of the air is lower than the WVP on the skin or respiratory tract of the animal [45]. For reference, the saturation WVP at the normal body temperature of a mouse (about 37°C) is 6.3 kPa [25]. The WVP was higher than 6 kPa in six of the reviewed studies, under which conditions the mice would not have been able to lose any heat by evaporation. Under those conditions, the air temperature exceeded the body temperature of the mouse, so it would not have been able to lose heat by any route. In those studies, the object was to induce hyperthermia in the mice.

### 4.4 Enhancing Relevance: Modeling Heat Impacts Across Populations

The broader context of the experimental designs in the reviewed studies reveals substantial limitations and opportunities for future research to enhance translational relevance to human health. A striking observation was the predominant focus on male physiology, which is incongruent with contemporary recommendations for research practice. Beyond reproductive health, it is essential to investigate outcomes in both sexes. Conversely, a number of studies that examined physical exertion during exposure to heat used only female mice [46, 47, 48, 49] as previous work had highlighted that male mice do not tolerate the same level of heat load or exercise duration as females [37]. This raises interesting translational implications for the human context.

Among studies that measured body temperature, half implemented heat‐exposure protocols that induced an increase of more than 4°C–7°C above baseline core temperature, representing extreme hyperthermia. The duration of heat exposure also warrants scrutiny; weeklong or longer exposures in mice can provide insight into the absolute limits of physiological systems but have limited translational utility, as humans will rarely be exposed to extreme conditions continuously. The timing of exposure during pregnancy and lactation adds further complexity, as the rapid and differential developmental timelines of the mouse fetus and neonates must be considered when extrapolating results to human outcomes.

The health context that we have addressed in this review highlights areas for future research. Mouse models could be leveraged to explore mechanistic drivers and interventions for key heat‐health concerns in humans, including impacts on older adults, children, and pregnant or lactating individuals; medication interactions; sleep; chronic disease; and mental health outcomes. Within the timeframe of the studies that are included in this review, only a few addressed these priority contexts. Finally, heat acclimation status, shaped by climate‐zone adaptations and air‐conditioning use, should be considered, as it influences physiological responses to extreme heat and is difficult to assess comprehensively in human studies.

### 4.5 Future Experimental Design Recommendations

To enhance the physiological relevance and translational value of heat‐exposure studies in mice, several aspects of experimental design warrant careful consideration. Foremost, researchers should clearly define the research question, including the specific human population the study is intended to inform (e.g., older adults, pregnant individuals, outdoor workers, or athletes), to ensure that experimental conditions appropriately model the relevant exposure patterns and physiological context. When the overarching objective is to improve understanding of human health impacts, study design should be deliberately aligned with this goal. Central to this process are animal welfare and ethical requirements, including adherence to institutional standards for mouse housing, such as group housing and provision of nesting materials. Within these frameworks, researchers should establish housing conditions that promote thermoneutrality, which include: (1) maintaining ambient temperatures around 26°C, (2) providing abundant bedding and nesting material (particularly for females), (3) controlling housing density to minimize cold‐induced thermogenesis; thereby allowing mice a more active role in thermoregulation under conditions more comparable to humans. Beyond control housing, the timing, severity, and duration of heat exposure should reflect realistic exposure for the human health context the study is modeling, as well as circadian biology, developmental, and organ‐specific considerations. Environmental factors such as group housing density, nesting material, and humidity should be consistently reported to enhance reproducibility. Finally, unless outcomes are explicitly sex‐specific, the physiological effects of heat exposure should be assessed in both sexes to capture potential sex‐dependent responses.

To address these gaps, a set of reporting items that we consider to be essential for heat‐exposure studies in mice is proposed, with a rationale and the frequency of such reporting in the studies covered by this review (Table 1). Further, researchers should carefully consider common sources of bias, such as randomization, blinding, and housing variables, and report these aspects in alignment with established risk‐of‐bias frameworks. This level of rigor is crucial to enhance the reliability and validity of preclinical research. To advance this agenda, physiology journals could consider the provision of formal guidance to authors on these issues for publication.

**TABLE 1:** Recommendations for minimum reporting requirements for mouse heat‐exposure studies.

#	Item to report	Rationale	% of studies reporting these items
**Housing environments**			
1	Ambient temperature of control and exposed housing (°C), with explicit statement of relationship to the mouse TNZ	Housing temperature is an important determinant of physiological and metabolic parameters and is essential to interpret the magnitude and direction of the heat‐induced effect [11, 22, 50]	95% reported ambient temperature, 4% referred to TNZ
2	Humidity or water vapor pressure	Humidity modulates evaporative heat loss. Two studies at the same ambient temperature can produce very different thermal outcomes depending on humidity; without it, the heat exposure ramifications are unclear [20, 51]	58%
4	Cage density (number of mice per cage), whether singly or group housed	A single‐housed mouse and group‐housed mice at the same ambient temperature experience different thermal loads (huddling shares heat and reduces heat loss) and different baseline stress states (isolation is itself a stressor) [31]	26%
5	Bedding type and depth, and nesting material provided	Nest building allows behavioral thermoregulation [52]	~10%
6	Light–dark phase during which heat was applied, with justification	As mice are nocturnal, researchers should consider the alignment of heat exposure with their active phase to better translate to human context [38].	45% reported phase of exposure, 14% exposed in the dark phase
**Animal characteristics**			
7	Strain, sex, age, and sample sizes of both control and heat‐exposed groups	Basic experimental variables [24, 53].	96%, 92%, 86%, 98%
**Body temperature measurement**			
8	Report core body temperature change due to heat exposure	Indicates severity of heat exposure, enabling translation to human considerations to be placed into context	65%
9	Method of body temperature measurement (rectal probe, intraperitoneal telemetry, body surface, etc.)	Each method of body temperature measurement is distinct; therefore, it should be considered when comparing results between studies [54]	65%
**Risk of bias and rigor**			
10	Randomization procedure for allocation to groups	Reduces selection bias [21].	64%
11	Blinding of outcome assessors	Reduces detection bias [21].	17%
12	A priori sample size calculation	Avoids underpowered studies [21].	5% did a priori sample size calculation
13	Compliance with animal welfare regulations and ethical approval	Required for the ethical conduct of animal research [21].	94%

## 5 Conclusion

Although the limitations of housing mice at standard laboratory temperatures have been recognized for many years, progress in adapting experimental models has been slow. The outcome of this review has broad implications across biomedical research but is particularly pressing given the growing interest in our attempts to understand the physiological impacts of heat. To ensure meaningful translation to human health contexts, there is an urgent need to re‐evaluate how mouse models of heat exposure and their controls are constructed. Future work should include a focused systematic synthesis of specific physiological outcomes from heat‐exposure studies in mice, with a re‐evaluation of individual findings against the methodological confounds that we have identified in this review. Through this systematic review, we highlight the critical gaps in current approaches and emphasize the need for more physiologically and translationally relevant experimental designs.

## Author Contributions

**Amina H. Rhaman:** conceptualization, investigation, writing – review and editing, data curation, writing – original draft, methodology, formal analysis. **Leaf R. Kardol:** conceptualization, investigation, writing – review and editing, writing – original draft, methodology, data curation, formal analysis. **Elizabeth Sorial:** investigation, data curation, methodology, writing – review and editing. **Caitlin S. Wyrwoll:** conceptualization, funding acquisition, investigation, methodology, supervision, writing – original draft, formal analysis. **Shane K. Maloney:** conceptualization, investigation, writing – review and editing, formal analysis, supervision, methodology, visualization. **Erin Kelty:** investigation, methodology, data curation, writing – review and editing. **Shannon Morgan:** investigation, data curation, methodology, writing – review and editing. **Danielle J. Russell:** investigation, data curation, methodology, writing – review and editing. **Ebony Quintrell:** investigation, writing – review and editing, methodology, data curation.

## Funding

This work was supported by the Wellcome Trust (227174/Z/23/Z). Caitlin S. Wyrwoll and Erin Kelty are supported by Fellowships from the Stan Perron Charitable Foundation. Amina H. Rhaman, Leaf R. Kardol, Ebony Quintrell, Danielle J. Russell, and Elizabeth Sorial are supported by an Australian Government Research Training Program Scholarship at The University of Western Australia. Leaf R. Kardol is supported by a Graduate Women Western Australia Scholarship. This work is related to funding from the National Health and Medical Research Council Special Initiative in Human Health and Environmental Change (Grant no. 2008937).

## Ethics Statement

The authors have nothing to report.

## Consent

The authors have nothing to report.

## Conflicts of Interest

The authors declare no conflicts of interest.

## Supporting information


**Figure S1:** Distribution of control housing temperatures in studies of heat exposure in mice, stratified by sex of the animals. Studies that did not report a numeric control housing temperature, or that did not report the sex of the animals, are excluded from this figure (*n* = 23).

**Data S2:** Data extracted from studies included in the review, including author name, year of publication, doi, title, country, aim, strain of mouse studied.

## Data Availability

The data that supports the findings of this study are available in the Supporting Information of this article.
