# Supplementary material for: Under Control? Mouse Models of Heat Exposure: A Systematic Review of Experimental Design and Methodology
Source: Acta Physiol (Oxf). 2026 Jul 23;242(8):e70281. doi: 10.1111/apha.70281 (PMC13396153; doi:10.1111/apha.70281)
Supplement: Supplementary file 1 — Figure S1: Distribution of control housing temperatures in studies of heat exposure in mice, stratified by sex of the animals. Studies that did not report a numeric control housing temperature, or that did not report the sex of the animals, are excluded from this figure (n = 23). [file APHA-242-e70281-s002.docx]

**Under control? Mouse models of heat exposure: a systematic review of experimental design and methodology.**

**Supplementary Material 1**

Amina H Rhaman^1^, Leaf R Kardol^2^, Shane K Maloney^2^, Ebony Quintrell^1,3,^ Elizabeth Sorial^1^, Shannon Morgan^4^, Danielle J Russell^1^, Erin Kelty^1^, and Caitlin S Wyrwoll*^2^

1 School of Population & Global Health, University of Western Australia, Perth, WA;

2 School of Human Sciences, University of Western Australia, Perth, WA;

3The Kids Research Institute Australia, Nedlands, WA;

4Medical School, University of Western Australia, Perth, WA

**Corresponding author: Caitlin Wyrwoll*

School of Human Sciences

The University of Western Australia

35 Stirling Highway

Crawley WA 6009

Email: caitlin.wyrwoll@uwa.edu.au

**Contents:**

**Supplementary Figure S1.** Distribution of control housing temperatures in studies of heat exposure in mice, stratified by sex of the animals. Studies that did not report a numeric control housing temperature, or that did not report the sex of the animals, are excluded from this figure (n = 23).

**Inclusion and Exclusion criteria**

**Search strategy**


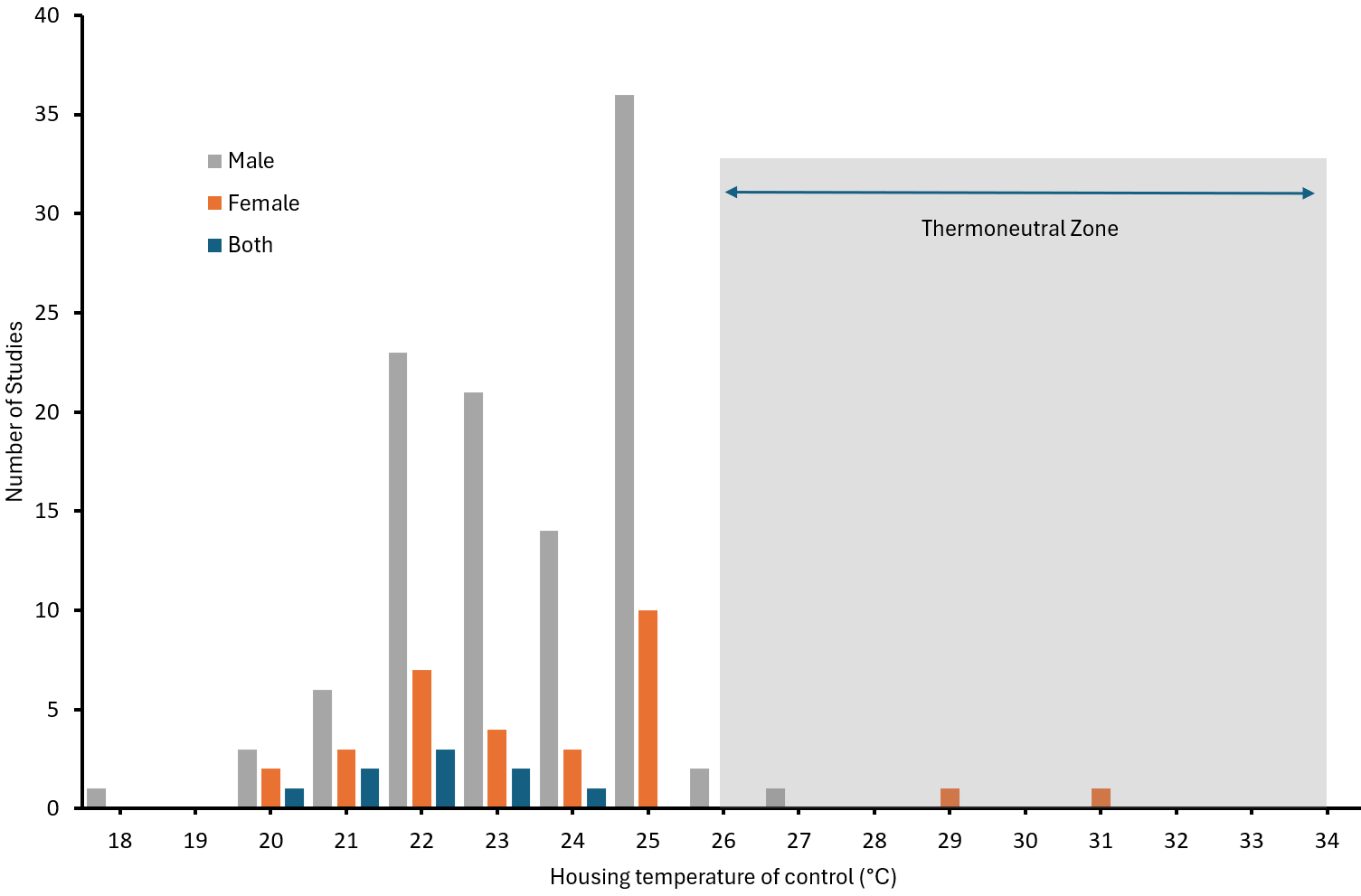


**Figure S1. Distribution of control housing temperatures in studies of heat exposure in mice, stratified by sex of the animals. Studies that did not report a numeric control housing temperature, or that did not report the sex of the animals, are excluded from this figure (n = 23).**

Inclusion/exclusion criteria

|  | Inclusion | Exclusion | Reason |
| --- | --- | --- | --- |
| Population | - Mice (Mus musculus) | - Mammals that are not mice (Mus musculus) - Non-mammalian species - Isolated cell and organ studies | - The focus is specifically on mouse models, a common experimental model for ascertaining the impact of environmental exposures on bodily systems. |
| Exposure | - High ambient temperature, as described as high temperature, heat exposure, hot environment, or similar | - Cold or warm ambient temperature - Exercise-related elevated body temperature will be excluded - Induction of fever - Exposure to hot baths - Heat exposure for the sole purpose of seizure induction | - This review prioritises investigating ambient heat-related exposures in mouse experimental models. Exposures to “warm” temperatures, or to heat for the purpose of seizure induction rather than as an exposure in its own right are not relevant. |
| Comparison | - Mice that **are not** exposed to a significant amount of heat (control group) | - Studies that do not include a comparison group or control group. | - Including studies with well-defined comparison groups allows for a clearer understanding of the specific impacts of ambient heat exposure by directly comparing outcomes with those not exposed to such conditions. |
| Outcomes | - Studies reporting on physiological, behavioural or neurological outcomes through any stage of the mouse lifecourse. | - Studies that do not specifically assess health-related outcomes. - Outcomes not measured with standardised, validated tools. | - Focus is any kind of physiological, behavioural or neurological outcome from conception through to old age. |
| Study Designs/Types | - Peer-reviewed original research articles - Experimental studies - Publications published since 2020. | - Review articles, commentaries, editorials, and case reports. - Grey literature - Studies that do not directly address heat exposure or physiological outcomes. | - Including peer-reviewed original research articles ensures that the review is based on high-quality and reliable data. - The exclusion of review articles, commentaries, editorials, case reports, and grey literature maintains the focus on primary research data, while excluding studies that do not directly address the topic ensures relevance and specificity. - Confining the work from 2020 onwards enables focused context for contemporary scientific design. |

Search Strategy

Search terms for population: **mouse** AND Exposure: **heat exposure.** Search to be performed for abstract, title, and keyword fields (or database equivalents) and restricted from 2014 to current.

| **Mouse** | **Heat exposure** |
| --- | --- |
| **MeSH terms** | |
| Mice/ | Hot temperature/ or heating/ |
| **Key terms** | |
| **Mouse or mus musculus or mice or murine** | Heat stress or hot temperature or hyperthermia or ambient heat or heat stroke or heatstroke or hot environment or heat exposure or heat strain |
